# Anaplastic Lymphoma Kinase Inhibition Therapy for Hereditary Neuroblastoma

**DOI:** 10.1200/PO-24-00886

**Published:** 2025-04-28

**Authors:** Yaël P. Mossé, Grace Polkosnik, Jenny Pogoriler, Peter Mattei, Lisa J. States, John M. Maris

**Affiliations:** ^1^ Division of Oncology and Center for Childhood Cancer Research, Children's Hospital of Philadelphia, Philadelphia, PA; ^2^ Perelman School of Medicine at the University of Pennsylvania, Philadelphia, PA; ^3^ Department of Pathology, Children's Hospital of Philadelphia, Philadelphia, PA; ^4^ Department of Surgery, Children's Hospital of Philadelphia, Philadelphia, PA; ^5^ Department of Radiology, Children's Hospital of Philadelphia, Philadelphia, PA

## Introduction

Germline testing for hereditary cancer predisposition has become an integral component of precision medicine. The genetic basis of neuroblastoma, a heterogeneous childhood cancer arising from the developing sympathetic nervous system, can segregate in families as an autosomal dominant trait.^1^ The majority of familial cases are due to gain-of-function mutations in the anaplastic lymphoma kinase (*ALK*) oncogene.^2,3^ The demonstration that these same alterations are the most common mutations in sporadic forms of neuroblastoma^2-6^ led to the development of small molecular inhibition therapies that are showing benefit in clinical trials.^7^

While 10%-15% of patients with neuroblastoma harbor a germline mutation in a cancer predisposition gene,^8,9^ only 1%-2% have a family history of the disease. Familial neuroblastoma typically arises at an earlier age of onset and presents with multiple primary tumor sites. The disease has incomplete penetrance, and unaffected obligate carriers are often observed. Inactivating mutations in *PHOX2B*, a homeobox gene that is crucial for the development of the autonomic nervous system, have also been identified in a small subset, usually accompanied by other disorders of the neural crest.^10-12^ A genome-wide association study (GWAS) of a large cohort of sporadic neuroblastomas has uncovered numerous polymorphisms associated with the disease,^13-15^ and ongoing work seeks to understand how these variants influence the penetrance of Mendelian mutations in *ALK* and other cancer predisposition genes.

Cancer predisposition genes may provide therapeutic vulnerabilities, such as defects in DNA damage repair pathway genes like *BRCA1*, providing a synthetic lethal vulnerability to poly(ADP-ribose) polymerase inhibition.^16,17^ While most cancer-predisposing mutations render loss-of-function defects, gain-of-function mutations like those observed in *ALK* may provide direct therapeutic opportunities. Here, we report on a mother and daughter with neuroblastoma and a shared germline *ALK* R1275Q mutation who are in a durable remission after single-agent ALK inhibition therapy.

## Case Reports

Patient 1, a first-born 6-month-old girl was diagnosed with stage IV intermediate-risk neuroblastoma when she presented with jaundice and bilateral adrenal tumors. Tumor biology showed favorable histology, no evidence for *MYCN* amplification, and DNA index of one and no loss of heterozygosity at chromosomes 1p and 11q.^18^ She was treated with eight cycles of chemotherapy according to the Children's Oncology Group protocol,^19^ followed by gross total resection with a right adrenalectomy and left partial adrenalectomy 6 months after initial diagnosis. Surveillance imaging 6 months after surgery showed a new left paraspinal chest mass with elevated urinary catecholamines. She had a thoracoscopic gross total resection, and pathology showed mitotically active undifferentiated tumor. Her urinary catecholamines improved but remained elevated after this surgery.

Subsequent restaging showed a new T6-7 intercostal soft tissue mass with positive uptake on ^123^I-metaiodobenzylguanidine (MIBG) scintigraphy. Stem cells were harvested in anticipation of high-risk neuroblastoma therapy. Simultaneously, germline testing was performed and showed an *ALK* R1275Q mutation.^2^ The patient was enrolled on a phase 1 trial of the first-generation ALK inhibitor crizotinib.^20^ She had an early complete response and remained on study for eight cycles, at which point she was removed because of grade 3 elevated alkaline phosphatase levels. She continued crizotinib via compassionate access, maintained a complete and durable remission with no further toxicity, and continued therapy for 5 years. Patient 1 remains in remission now 8 years off-therapy and returns for whole-body magnetic resonance imaging (MRI) surveillance, urine catecholamine testing, and circulating tumor DNA (ctDNA) profiling every 6 months, which during the course of her therapy was shown to be a sensitive method to detect early relapse.^21,22^

**FIG 1.**
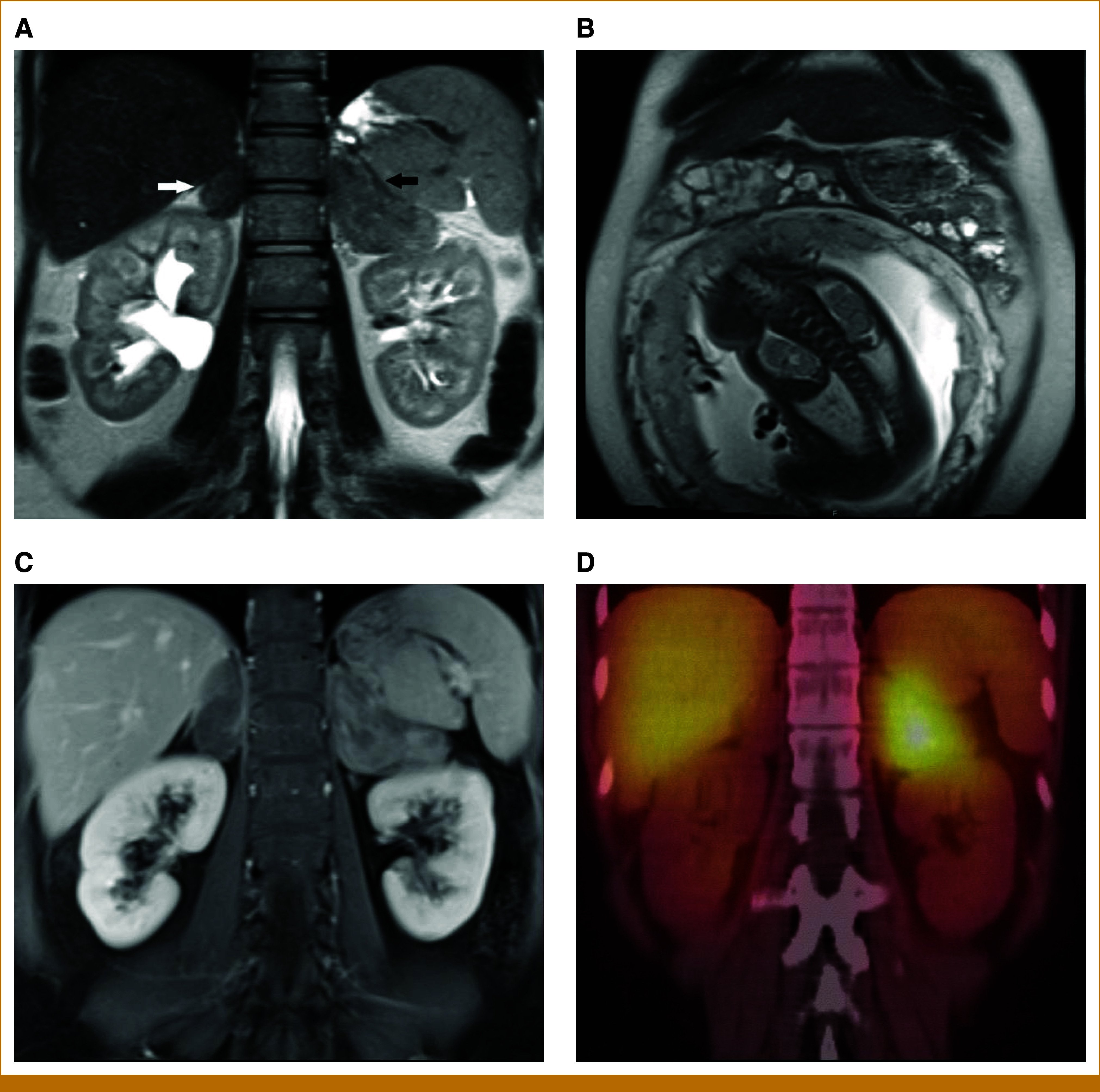
Radiographic findings. (A) Coronal T2-weighted image shows a small, ovoid right adrenal mass (white arrow) and larger, triangular left adrenal mass (black arrow) with heterogeneity and higher T2 signal intensity than the right adrenal mass. (B) Coronal T2 HASTE fetal MRI image shows a fetus with normal adrenal glands. (C) Two months later, postpartum, a contrast-enhanced coronal T1-weighted image with fat saturation shows asymmetric enhancement, mild and homogeneous on right and moderate and heterogenous on left. (D) Coronal I-123MIBG SPECT/CT-fused image shows no uptake in right adrenal mass and moderate uptake in left adrenal mass. MIBG, metaiodobenzylguanidine; SPECT/CT, single-photon emission computed tomography/computed tomography.

Patient 2, the 36-year-old healthy mother of patient 1 was found to have the same heterozygous germline ALK R1275Q mutation identified through genetic testing. Genetic counseling was provided, and surveillance imaging was recommended but not pursued. When the patient became pregnant 5 years after the birth of patient 1, chorionic villus sampling was performed and showed that the *ALK* R1275Q-mutant allele was not inherited by the fetus. Subsequently, the mother presented at 29 weeks of gestation with subacute left flank pain. An ultrasound showed enlarged bilateral adrenal glands, and an MRI confirmed the presence of bilateral adrenal tumors (Figs 1A-1C). Recommendation was made to deliver the fetus followed by staging of the patient. Examination of the placenta revealed no evidence of tumor, and the premature baby boy was healthy. Postnatal evaluation showed that the left adrenal tumor was MIBG-avid, whereas the right was not and there was no evidence of disease elsewhere (Fig 1D). Urine catecholamines were elevated, and no tumor biopsy was performed.

**FIG 2.**
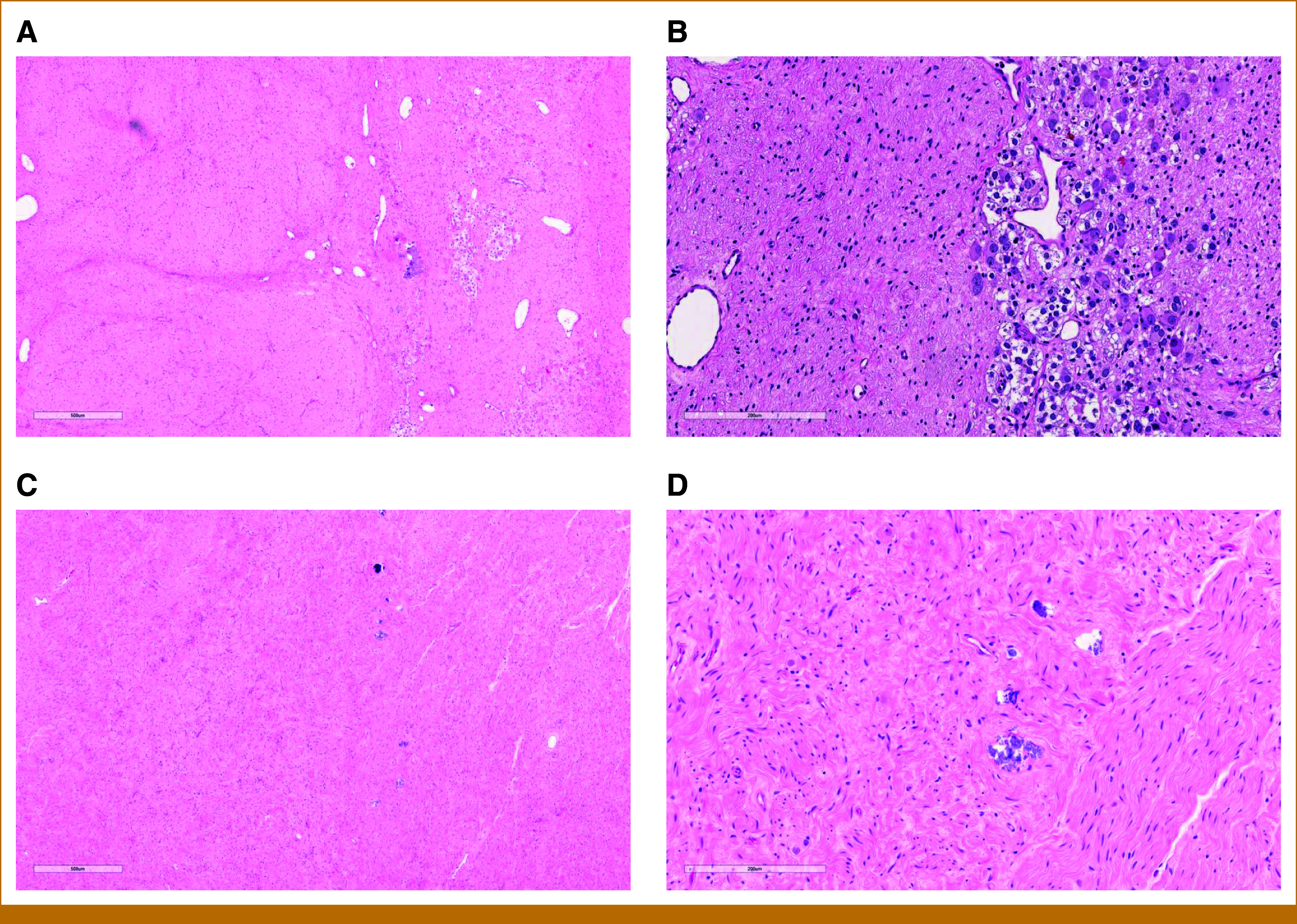
Pathology. The right adrenal showed a (A) hypocellular spindle cell lesion with rare ganglion cells and (B) calcifications identified at higher power. The left adrenal showed a (C) similar low-power spindle cell lesion, (D) but islands of residual neuroblastoma can be appreciated with neuropil and neuroblasts, some of which are differentiating.

Treatment with crizotinib was initiated 5 days after delivery. One month later, MRI and MIBG scans showed stable disease, and urinary catecholamines had normalized. Because of difficulties in tolerating therapy, crizotinib was replaced with alectinib. She underwent laparoscopic surgery to remove the bilateral adrenal tumors. The left adrenal tumor was well-vascularized but amenable to a gross total resection with preservation of a portion of the left adrenal gland. The right-sided tumor was less-well vascularized and nearly completely resected, leaving the right adrenal gland preserved. Pathology showed that the left adrenal mass was composed of predominantly Schwannian-type stroma with intermixed ganglion cells and calcifications (Fig 2A). Small islands of neuropil with differentiating neuroblasts remained (Fig 2B). Although classification of neuroblastoma is usually not performed after treatment, the morphology was compatible with ganglioneuroblastoma, intermixed. The right adrenal mass was composed entirely of Schwannian-type stroma with rare ganglion cells and scattered calcifications with no neuroblastic foci present (Figs 2C and 2D). Postoperative images indicated resolution of MIBG-avid disease in the left adrenal bed. Three months after resection, MRI showed no evidence of disease. The patient continued ALK inhibition with alectinib for 28 months after a complete response was achieved. She remains in a complete remission and returns with her daughter for surveillance every 6 months. ctDNA evaluations for both patients consistently show an expected *ALK* R1275Q variant allele frequency of 50%.

The authors of this study obtained verbal consent from the patients described in this case report to have their anonymized information published in the medical literature. Parents provided informed consent, and assent was obtained per institutional guidelines. The participant was not compensated. Local institutional review board was notified of all serious, unexpected adverse drug reactions involving risk to human patients.

## Discussion

Neuroblastoma pedigrees show notable heterogeneity, with both benign and malignant forms occurring in the same family. This report describes a case of multifocal neuroblastoma in an infant with no family history of the disease that was refractory to standard therapy. Genetic testing was performed based on the presentation of bilateral adrenal tumors, and she was found to harbor a germline *ALK* R1275Q mutation as a toddler. Perhaps germline testing should have been considered earlier, given her presentation, but the timing coincided with the initial discovery of germline mutations in neuroblastoma, and awareness was just emerging. Her then 36-year-old mother was found to be an unaffected carrier of the same mutation. As surveillance imaging was not pursued after initial genetic counseling, it is impossible to know if the mother developed adrenal tumors earlier in life that spontaneously differentiated, as is known to occur with biologically favorable neuroblastomas,^23,24^ or if pregnancy itself triggered tumorigenesis. The rationale for continuous ALK inhibitor treatment after biochemical response to crizotinib and gross total surgical resection of both adrenal tumors showing favorable histology was solely based on the presence of a germline *ALK* mutation. Their cases illustrate the highly variable tumorigenesis timeline of ALK-driven familial neuroblastoma, perhaps supported by the nonconcordance of MIBG avidity of the tumors, as well as the sensitivity to ALK inhibition therapy. The initial discovery of germline *ALK* mutations raised a number of questions, including the role of pharmacologic inhibition and whether this could be toxic rather than beneficial in this context.^25^ There are case reports of pregnancy while receiving ALK inhibition, demonstrating that exposed infants appear free of adverse health effects, with little known about risks of breastfeeding.^26-28^ Patient 1, who started ALK inhibition therapy at age 18 months and continued for 5 years, has no late effects and is a vibrant teenager.

This family demonstrates that patients with germline *ALK* mutations have a lifelong risk of developing new neuroblastic tumors and thus require surveillance through adulthood, similar to Li-Fraumeni syndrome.^29^ With the advent of ctDNA and whole-body MRI surveillance,^21,22,30^ precise monitoring is now possible to document molecular remission and surveil for recurrence. Because of the rarity of familial neuroblastoma, guidance for surveillance when a germline *ALK* mutation is identified is still evolving. *ALK* mutations differ in their biochemical autoactivation of the kinase, and the most activating mutations (eg, F1174 and F1245 residues) are not tolerated in the germline.^31^ Thus, penetrance likely differs not only based on the exact mutation but also by polygenic risk of common variants discovered through GWAS efforts.^13-15^ Patient 2 (and many others in our clinical experience) shows that late-onset neuroblastoma in those harboring a germline ALK mutation is clearly possible. This is in contrast to recent recommendations suggesting that surveillance should be performed for only the first decade of life^32^ since 98% of sporadic neuroblastomas are diagnosed before age 10 years.^33^ We recommend revising this guidance and surveilling these patients with ctDNA and whole-body MRIs annually as more data can be gathered. We posit that ctDNA is more powerful for detection of new disease and guiding patient management than MRI and will become the standard of care in the future.

Finally, these two cases raise new questions about the impact of germline susceptibility on clinical management, duration of therapy, and outcome. We postulate that these patients are less likely to develop acquired resistance compared with patients with somatic mutations who have branched genomic events and intratumor heterogeneity. Conversely, the observation of different pathologies in the distinct bilateral tumors suggests some level of heterogeneity. Patients with a germline *ALK* mutation should be offered ALK inhibition therapy as the primary treatment modality, perhaps even as monotherapy, and the role of surgery and chemotherapy should be reconsidered. In addition, genetic screening should be offered in the absence of a family history, and indefinite longitudinal surveillance should be recommended for those affected with the disease, as well as for silent carriers, especially as we refine the merits of biochemical end points like serial ctDNA as the primary surveillance tool.
